# Evaluation of the relationship between IL-12, IL-13 and TNF-α gene polymorphisms with the susceptibility to brucellosis: a case control study

**DOI:** 10.1186/s12879-019-4678-8

**Published:** 2019-12-09

**Authors:** Sima Kazemi, Asad Vaisi-Raygani, Fariba Keramat, Massoud Saidijam, Ali Reza Soltanian, Mahdi Alahgholi-Hajibehzad, Seyed Hamid Hashemi, Mohammad Yousef Alikhani

**Affiliations:** 1 Department of Microbiology, School of Medicine, Hamadan University of Medical Sciences, Hamadan, Iran; 2 Department of Clinical Biochemistry, School of Medicine, Kermanshah University of Medical Sciences, Kermanshah, Iran; 3 Brucellosis Research Center, Hamadan University of Medical Sciences, Hamadan, Iran; 4 Research Center for Molecular Medicine, Hamadan University of Medical Sciences, Hamadan, Iran; 5 Department of Biostatistics and Epidemiology, School of Public Health, Hamadan University of Medical Sciences, Hamadan, Iran; 6 Department of Immunology, Hamadan University of Medical Sciences, Hamadan, Iran

**Keywords:** Cytokines, Polymorphisms, Brucellosis, Iran

## Abstract

**Background:**

The cytokine gene polymorphism is important for the genetic susceptibility of infectious diseases. The aim of the present study was to investigate the relationship between TNF-α, IL-12, and IL-13 gene polymorphisms and predisposition to brucellosis.

**Methods:**

In this study, 107 patients with brucellosis and 107 healthy individuals were evaluated. The SNPs of TNF-α)- 238 G/A) and IL-12 (+ 1188 A/C) were done by amplification refractory mutation system-polymerase chain reaction (ARMS-PCR) and IL-13 genotyping at positions − 1512 (A/C) and − 1112 (C/T) were analysis by restriction fragment length polymorphism-polymerase chain reaction (RFLP-PCR) methods. IL-12, IL-13 and TNF-α serum levels were measured by a sandwich enzyme-linked immunosorbent assay (ELISA).

**Results:**

IL-13 (−1512A/C) was associated with Brucellosis risk in dominant model (OR (95% CI) = 2.17 (1.02–4.62)), *P-value* = 0.041. However, there was no difference in allele and genotype frequencies of TNF-α)- 238 G/A), IL-12 (+ 1188 A/C) and IL-13 [− 1512 (A/C) and − 1112 (C/T)] between patients and controls. Serum levels of IL-12 and TNF-α were significantly more frequent in the patients than in the control groups.

**Conclusions:**

The IL-13 gene polymorphism can be used as a biomarker for detecting susceptibility to Brucella disease.

## Background

*Brucella spp.* are Gram-negative, non-spore forming, non-motile, facultative intracellular bacteria, which cause an infectious disease called Brucellosis [1]. Transmission of these bacteria to humans through the use of unpasteurized dairy products and inhalation of contaminated aerosols with infected animal [2, 3]. Clinical features of active brucellosis in humans included fever, sweating, weight loss, arthralgia, hepatomegaly, splenomegaly, headache, endocarditis and clinical manifestations in domestic animal in both female and males include abortion, vaginal secretions, placenta retention, low fertility rate, epididymitis, infertility, sperm abnormalities. Abortion often has symptoms of placental retention and metritis that may cause infertility [4–7]. The disease in areas like the Middle East, Eastern Europe, Africa, Latin America is endemic [8, 9].

Hamadan province is one of the high prevalent cities in western Iran [10]. Cytokines set the pathway for adaptive immune responses [11]. Studies have shown that reducing or increasing the expression of cytokines can play a major role in pathogenicity. Moreover, protection in this disease is carried out by T-Helper 1 cells (Th_1_) while T-Helper 2 cells (Th_2_) response is effective in exacerbating the disease [12]. Upon infection, phagocytes are activated to produce proinflammatory cytokines including Tumor necrosis factor-a (TNF-a) and Interleukin-12 (IL-12). Interleukin 12 to produce interferon gamma, which stimulates the response of Th_1_ and activates macrophages. Activated macrophages can kill intracellular *Brucella* and eliminate the infection [13, 14]. Similar IFN-γ, TNF-a as well as is a vital agent for the clearance of brucellosis infection from the host [15]. IL-12, is one of the inherent immune inflammatory cytokines, plays a major role in controlling infection in intracellular bacteria. This cytokine is a central cytokine in the differentiation of Th_1_ cells [16]. IL-13 is pivotal pro-inflammatory cytokines and down-regulates Th_1_ responses. Consequently this cytokine promotes intracellular infection [17]. Genetic variation in interleukin 12, interlukin-13 and TNF-α has been studied in various diseases including mycobacterial infection, type1diabetes, periodontitis, viral diseases and autoimmune disorders [18–22]. Polymorphisms in the genes of cytokines can increase or decrease their expression and affect the determination of acute or chronic disease [23]. Since genetic variation in various populations and the presence of specific polymorphisms in patients, understanding the cytokine pattern as an important factor in the clinical outcome of brucellosis infection can be effective in controlling the disease. The aim of the present study was to investigated the association between TNF-α)- 238 G/A), IL-12 (+ 1188 A/C), and IL-13 (− 1512 A/C and − 1112 C/T) gene polymorphisms and their serum levels and susceptibility to brucellosis comparison to healthy subjects.

## Methods

### Patients and controls

This study was performed in the Infectious Diseases Unit at Sina Hospital of Hamadan province, Iran. Between December 2017 and June 2018. The present study included 107 patients (79 men and 28 women) with brucellosis (age range 17–78 years and mean ± SD = 43.63 ± 16.21) and 107 healthy individuals as a control group (76 men and 31 women, age range 20–60 and mean ± SD = 36.37 ± 9.11). Diagnosis of brucellosis was based on clinical findings, positive serological tests and positive blood cultures or PCR [24].

Inclusion criteria for healthy people included no previous contact with animals, no consumption of unpasteurized dairy products and without any clinical symptoms and exclusion criteria were any antibody to brucellosis in serological tests. Healthy individuals were chosen from the same geographical areas of the patients.

The Ethical Committee of Hamadan University of Medical Sciences evaluated and approved the investigation and written informed consent was obtained from all participants (Ethical committee ID: IR.UMSHA.REC.1396. 157).

### DNA isolation and cytokine genotyping

Genomic DNA was extracted from blood samples by blood DNA extraction kit (Sina- Clon, Iran) according to the manufacturer’s protocol. The IL-12 (+ 1188 A/C) and TNF-α (− 238 A/G) genotyping was carried out by amplification refractory mutation system polymerase chain reaction method (ARMS-PCR) technique.

The primers used in the PCR reaction for IL-12 included common primer 5’ATCTTGGAGCGAATG GGC at 3′, C allele primer 5’TTGTTTCAATGAGCATTTAGCATCT 3’and A allele primer 5’TTG TTTCAATGAGCATTTAGCATCG 3′. The thermocycling conditions for IL-12 included a primary denaturation step at 94 °C for 3 min followed by 35 cycles at 94 °C for 30 s, annealing temperatures at 65 °C for 45 s, extension at 72 °C for 45 s and a final extension step in 72 °C for 5 min. The PCR products for IL-12 were 784 bp.

The primers used in the PCR reaction for TNF-α (− 238 A/G) included common primer 5’CCGGATCATGCTTTCAGTGC 3′, G allele primer 5’AGACCCCCCTCGGAATCG 3′, and A allele primer 5’AAGACCCCCCTCGGAATCA 3′. The forward and reverse primers for internal control (*B2-microglobulin* gene) were 5’CCAAAGATTCAGGTTTACTCACG 3′, and 5’ACTTAACTATCTTGGGCTGTGAC3′ respectively with cycling conditions of 95 °C 4 min, followed by initial DNA denaturation at 95 °C for 4 min, then 25 cycles of denaturation at 95 °C for 20 s, annealing temperature for TNF-α (− 238 A/G) and *B2-microglobulin* gene at 58 °C and 66 °C respectively for 50 s and extension at 72 °C for 50 s, followed by the final extension at 72 °C for 10 min. The PCR products for TNF-α and *B2-microglobulin* were 590 bp and 266 bp respectively [25, 26].

The IL-13 (−1512A/C) and IL-13 (−1112C/T) SNPs were analyzed by the polymerase chain reaction-restricted fragment length polymorphism (PCR-RFLP) method.

For IL-13 (−1512A/C), the primers were 5’CAACCGCCGCGCCAGCGCCTTCTC 3′ and 5’CCGCTA CTTGGCCGTGTGACCGC 3′, and the restriction enzyme was BstUI (Fermentase, Lithuania). The A allele yielded 2 bands of 216 and 29 bp and C allele yielded 194, 29, and 22 bp fragments. For IL-13 (^_^1112C/T), the primers were 5’GGAATCCAGCATGCCTTGTGAGG 3’and 5’GTCGCCTTTTCCTGCTCTTCCCGC 3’and the restriction enzyme was BstUI; the digestion revealed the fragments of 223 and 23 bp for C allele and 246 bp for the T allele.

PCR program for IL-13 (−1512A/C and − 1112C/T) was applied under the following conditions: Initial denaturation at 94 °C, 12 min; followed by 35 cycles of denaturation at 95 °C, 30 s; annealing for 2 min at 65 °C for IL-13 (−1512A/C); 54 °C for IL-13 (−1112C/T) and extension at 72 °C for 40 s followed by a final extension step at 72 °C for 5 min [27]. PCR products were separated on 2% agarose gel contained 0.5 μg/ mL SYBR Safe and photographed under UV light.

### Determination of cytokine levels

Three milliliter of peripheral venous blood sample taken from all patients and control groups. The blood samples were centrifuged at 3000 rpm for 7 min; the sera were stored at − 80 °C until assayed.

Serum cytokine levels (IL-12, IL-13, TNF-α) were analyzed by commercially available ELISA kits (Invitrogen- Thermo Fisher Scientific, Vienna, Austria) according to the manufacturer’s instructions.

### Statistical analysis

An online program, SNPSTats (http://bioinfo.iconcologia.net/ SNPstats) was used to calculate the genotype frequencies, Hardy Weinberg Equilibrium (HWE) and odds ratios. Version 16 of SPSS was used for analysing the data. *p value* less than 0.05 was considered to be statistically significant.

## Results

Table 1 shows the genotype and alleles of study participants. IL-13 (− 1512 A/C) was associated with brucellosis risk in dominant model (OR (95% CI) = 2.17 (1.02–4.62)), *P* = 0.041). Allele and genotype frequencies of IL12 (+ 1188 A/C), IL-13 (− 1112 C/T), IL-13 (− 1512 A/C) and TNF-α (− 238 G/A) were not significantly different between patients and controls. The serum levels of IL-12, TNF-α and IL-13 in patients with brucellosis and in controls are shown in Table 2.

**Table 1:** Association analysis of TNF-α, IL-12 and IL-13 gene polymorphisms and brucellosis

SNP	Model	Genotype	Patients no (%)	Controls no (%)	OR (95% CI)	*P-value*
TNF-α (− 238 G/A)		G/G	10 (9.3)	13 (12.2)	1.00	0.51
		A/G	97 (90.7)	94 (87.8)	0.75 (0.31–1.78)	
		A/A	0	0	–	
	Allele	A	97 (45)	120 (56)	0.927 (0.633–1.358)	0.697
		G	117 (55)	94 (44)		
IL-12 (+1188 A/C)	Codominant	C/C	22 (20.6)	29 (27.1)	1.00	0.19
		A/C	78 (72.9)	66 (61.7)	0.64 (0.34–1.22)	
		A/A	7 (6.5)	12 (11.2)	1.30 (0.44–3.85)	
	Dominant	C/C	22 (20.6)	29 (27.1)	1.00	0.26
		A/C-A/A	85 (79.4)	78 (72.9)	0.70 (0.37–1.31)	
	Recessive	C/C-A/C	100 (93.5)	95 (88.8)	1.00	0.23
		A/A	7 (6.5)	12 (11.2)	1.80 (0.68–4.78)	
	Overdominant	C/C-A/A	29 (27.1)	41 (38.3)	1.00	0.08
		A/C	78 (72.9)	66 (61.7)	0.60 (0.34–1.07)	
	Allele	A	126 (59)	112 (52)	0. 962 (0.656–1.412)	0.845
		C	88 (41)	102 (48)		
IL-13 (−1112C/T)	Codominant	T/T	22 (20.6)	12 (11.2)	1.00	0.15
		C/T	80 (74.8)	91 (85)	2.09 (0.97–4.48)	
		C/C	5 (4.7)	4 (3.7)	1.47 (0.33–6.52)	
	Dominant	T/T	22 (20.6)	12 (11.2)	1.00	0.06
		C/T-C/C	85 (79.4)	95 (88.8)	2.05 (0.96–4.39)	
	Recessive	T/T-C/T	102 (95.3)	103 (96.3)	1.00	0.73
		C/C	5 (4.7)	4 (3.7)	2.05 (0.96–4.39)	
	Overdominant	T/T-C/C	27 (25.2)	16 (14.9)	1.00	0.059
		C/T	80 (74.8)	91 (85)	1.92 (0.97–3.82)	
	Allele	T	124 (58)	115 (54)	1.186 (0.810–1.738)	0.381
		C	90 (42)	99 (46)		
IL-13 (−1512A/C)	Codominant	A/A	23 (21.5)	12 (11.2)	1.00	0.094
		A/C	80 (74.8)	88 (82.2)	2.11 (0.99–4.51)	
		C/C	4 (3.7)	7 (6.5)	3.35 (0.82–13.78)	
	Dominant	A/A	23 (21.5)	12 (11.2)	1.00	0.041
		A/C-C/C	84 (78.5)	95 (88.8)	2.17 (1.02–4.62)	
	Recessive	A/A-A/C	103 (96.3)	100 (93.5)	1.00	0.35
		C/C	4 (3.7)	7 (6.5)	1.80 (0.51–6.35)	
	Overdominant	A/A-C/C	27 (25.2)	19 (17.8)	1.00	0.18
		A/C	80 (74.8)	88 (82.2)	1.56 (0.81–3.03)	
	Allele	A	126 (59)	112 (52)	0.767 (0.523–1.124)	0.174
		C	88 (42)	102 (48)

**Table 2:** Serum levels of TNF-α, IL-12 and IL-13 in patients with brucellosis and controls

Interleukin (pg/ml)	Patients Mean ± SD	Controls Mean ± SD	*p- value*
TNF-α	15.20 ± 60.37	1.34 ± 1.41	< 0.001
IL-12	4.39 ± 4.77	2.68 ± 2.02	< 0.001
IL-13	4.23 ± 13.95	2.30 ± 1.62	< 0.308

Serum level of IL-12 were 4.39 ± 4.77 pg/ml and 2.68 ± 2.02 in brucellosis and control groups, respectively, which shown a significant difference (*p* < 0.001). The mean serum TNF-α level of patients with brucellosis (15.20 ± 60.37 pg/ml) compared with the controls groups (1.34 ± 1.41 pg/ml). This difference was statistically significant (*p* < 0.001). Serum levels of IL-13 were (4.23 ± 13.95, 2.30 ± 1.62) pg/ml in brucellosis and controls, respectively. As shown in Table 3, no significant associations were found between serum levels of IL-13, TNF-α and IL-12 and gene polymorphisms [TNF-α (− 238 G/A), IL-12 (+ 1188 A/C), IL-13 (− 1112C/T) and IL-13 (−1512A/C)] in patients with brucellosis and controls. The AA, AC and CC genotype at positions IL-13 (−1512A/C) and IL-12 (+ 1188 A/C) showed a higher IL-13 and IL-12 serum levels in the case group than the control group. The AG and GG genotype at position TNF-α (− 238 G/A) demonstrated a higher TNF-α serum level in patient group than the control group. The patient group with IL-13(− 1112 C/T) CC, CT and TT genotypes had higher IL-13 serum level than that of the control group.

**Table 3:** Serum levels of TNF-α, IL-12 and IL-13 in brucellosis patients and control groups with different genotypes

SNPs	Groups/Genotypes	Serum levels				*p*- *value* ^b^
IL-13 (−1512A/C)		Serum IL-13 levels (pg/ml)				0.07
	Genotypes	AA	AC	CC	*p-value* ^a^	
	Case (*n* = 107)	2.29 ± 2.31	4.87 ± 16.06	2.47 ± 0.60	< 0.001	
	Control (*n* = 107)	1.89 ± 1.28	2.41 ± 1.68	1.62 ± 1.14	< 0.001	
	*p-value* ^C^	0.58	0.16	0.21		
IL-13(−1112 C/T)	Genotypes	CC	CT	TT		0.187
	Case (*n* = 107)	2.47 ± 1.62	4.59 ± 16.06	3.29 ± 3.08	< 0.001	
	Control (*n* = 107)	1.82 ± 1.27	2.34 ± 1.66	2.21 ± 1.46	< 0.001	
	*p-value* ^C^	0.24	0.19	0.26		
TNF-α (−238 G/A)		Serum TNF-α levels (pg/ml)				0.718
	Genotypes	AA	AG	GG		
	Case (*n* = 107)	–	15.71 ± 63.22	10.22 ± 16.50	< 0.001	
	Control (*n* = 107)	–	1.28 ± 1.39	1.72 ± 1.54	< 0.001	
	*p-value* ^C^	–	0.02	0.07		
IL-12 (+1188 A/C)		Serum IL-12 levels (pg/ml)				0.172
	Genotypes	AA	AC	CC		
	Case (*n* = 107)	2.71 ± 1.69	4.85 ± 5.47	3.28 ± 1.20	< 0.001	
	Control (*n* = 107)	2.60 ± 1.67	2.70 ± 2.10	2.66 ± 2.03	< 0.001	
	*p-value* ^C^	0.89	0.003	0.02		

^a^ *p-value* was calculated based on one-sample Chi-square test, the genotypes frequencies were compared in case and control separately

^b^ *p-value* was calculated based on Chi-square test in a contingency table

^c^ *p-value* was calculated based on independent t-test/Mann-Whitney test

The results shown that serum levels of IL-13 (−1512A/C), IL-13 (− 1112 C/T), TNF-α (− 238 G/A) and IL-12 (+ 1188 A/C), were higher in all genotypes of patients than in the control group. However, the serum level of TNF-α (− 238 G/A) genotype AG and serum level of IL-12 (+ 1188 A/C) genotypes CC and AC were significantly (*p-value* < 0.05) higher in patients with control groups (Table 3). Besides, there were significant association of IL-12 (+ 1188 A/C), TNF-α (− 238 G/A) and IL-13 (− 1512A/C) polymorphisms with the serum levels of these cytokines in both patient and healthy groups separately.

## Discussion

Numerous factors including immunity, environment, and genetics affect the susceptibility to infectious diseases. Studies have shown that single nucleotide polymorphism in genes can influence the regulation of immune responses. The aim of this study was to investigate the probable relationship between TNF-α)- 238 G/A), IL-12 (+ 1188 A/C), and IL-13 (− 1512 A/C and -1112 C/T) gene polymorphisms and their serum concentrations and susceptibility to Brucella infection.

Our results showed that there was no significant association between the genotypes or alleles of IL-13 (− 1112 C/T), IL-13 (− 1512 A/C), IL12 (+ 1188 A/C) and TNF-α)- 238 G/A) between patients with brucellosis and the control group, although the dominant model of IL-13 (− 1512 A/C) influence the susceptibility to brucellosis (*p value*: 0.041). The previous study have reported significant differences between patients with brucellosis and controls in GG/GG genotypes of TNF-α (− 238 G/A,-308 G/A) and G allele of TNF-α (− 238 G/A) locations [28]. According to study by Eskandari-Nasab et al. GG genotype at position (− 308 G/A) was higher in the control group than in the patients and this genotype was a protective factor while GA genotype, A allele, AAG haplotype were a risk factor against developing brucellosis [29]. Caballero et al. from Spain has shown that TNF-a (− 308) polymorphisms influence the susceptibility to brucellosis [15].

Studies on Interleukin 12 shown that the frequencies of IL-12 AA genotype and A allele were higher in controls than in patients [30, 31]. Previous studies suggested that IL-12 (+ 1188) was related to the susceptibility or resistance to brucellosis and tuberculosis [32, 33]. An additional study found no influence of IL-13 polymorphisms on brucellosis infection [34]. Cytokines play a key role in the regulation of the immune responses [35–37]. In the present study, patients had significantly elevated serum levels of IL-12 and TNF-α compared to healthy controls. In terms of IL-13 levels, no significant difference was found between patient and control groups. In accordance with our results, Sanaei Dashti et al. observed that TNF-α and IL-12 serum levels were elevated significantly in brucellosis patients compared to the levels in other febrile patients [38]. In addition, the results of Demirdag and colleagues showed that serum level of TNF- α was high in patients than in controls and reported that this cytokines involved in the pathophysiology of brucellosis [39]. Another study reported that serum level of IL-12 in acute and chronic brucellosis patients were significantly higher than control group [40]. In contrast to our study, Ahmed et al. demonstrated serum level of TNF- α was not detectable in all the serum samples collected from patients; while high serum levels of IL-12 in the patient group compared to the control and patients without brucellosis groups was reported [41]. In our study, no significant associations were found between serum levels of IL-13, TNF-α and IL-12 and different genotypes [TNF-α (− 238 G/A), IL-12 (+ 1188 A/C), IL-13 (− 1112C/T and IL-13-1512A/C)] of the brucellosis patients and controls.

We did not find any study of association between polymorphism IL-13 (− 1512 A/C) and infectious diseases in the literature except for brucellosis [34], although the polymorphism of IL-13 (− 1112 C/T) has been shown to be associated with susceptibility to periodontitis [42]. There were no studies about association between serum levels of these cytokines and gene polymorphisms in brucellosis.

## Conclusions

The IL-13 gene polymorphism can be used as a biomarker for detecting susceptibility to brucella disease. Further studies with higher sample size in different populations and the effect of response to treatment on cytokine concentrations should be considered.

## Data Availability

The datasets used and/or analyzed during the current study available from the corresponding author on reasonable request.

## Abbreviations

ARMS: Amplification refractory mutation system
ELISA: Enzyme-linked immunosorbent assay
IFN-γ: Interferon gamma
IL: Interleukin
PCR: Polymerase chain reaction
RFLP: Restricted fragment length polymorphism
TNF-α: Tumor necrosis factor alpha
